# Plastid-cytosol partitioning and integration of metabolic pathways for APS/PAPS biosynthesis in *Arabidopsis thaliana*

**DOI:** 10.3389/fpls.2014.00751

**Published:** 2015-01-22

**Authors:** Anne-Sophie Bohrer, Stanislav Kopriva, Hideki Takahashi

**Affiliations:** ^1^Department of Biochemistry and Molecular Biology, Michigan State UniversityEast Lansing, MI, USA; ^2^Botanical Institute and Cluster of Excellence on Plant Sciences, University of Cologne, CologneGermany

**Keywords:** sulfur metabolism, sulfate assimilation, subcellular localization, metabolic flux, metabolite distribution

## Abstract

Plants assimilate sulfate from the environment to synthesize biologically active sulfur-containing compounds required for growth and cellular development. The primary steps of sulfur metabolism involve sequential enzymatic reactions synthesizing adenosine 5′-phosphosulfate (APS) and 3′-phosphoadenosine 5’-phosphosulfate (PAPS). Recent finding suggests that an adenosine nucleotide transport system facilitating the exchange of PAPS and 3′-phosphoadenosine 5′-phosphate across the plastid envelope is essential for establishing an intimate connection between the plastidic and cytosolic sulfate assimilation pathways in plants. Subcellular partitioning and integration of metabolic pathways provide focal points for investigating metabolic flux regulations. This perspective article presents an integrative view of sulfur metabolic flux control mechanisms with an emphasis on subcellular partitioning of APS/PAPS biosynthetic pathways in *Arabidopsis thaliana*.

## SUBCELLULAR LOCALIZATION AND PATHWAY DISTRIBUTIONS

**FIGURE 1:**
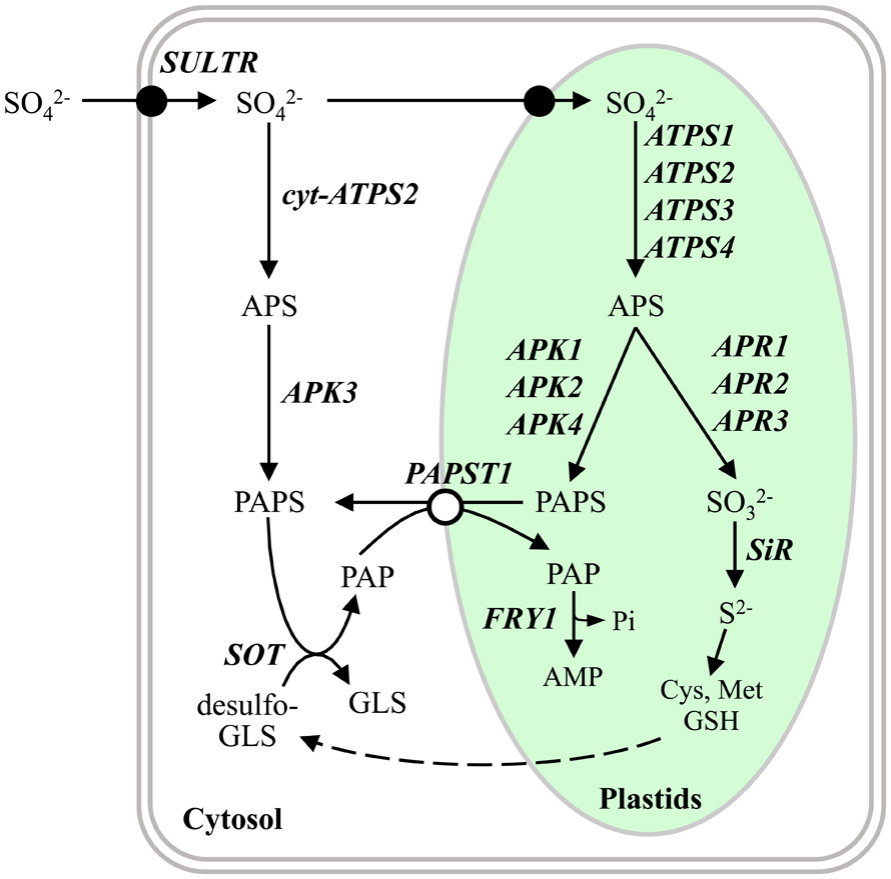
**Metabolic pathway of sulfur assimilation in *Arabidopsis*.** The illustration shows subcellular partitioning of sulfate assimilation and APS/PAPS metabolic pathways in plastids and cytosol in *Arabidopsis*. Cys, Met, and GSH biosynthetic pathways are simplified in this diagram to describe their connections downstream of the reductive sulfate assimilation pathway. The names of enzymes and transporters are indicated in bold italicized letters. Closed and open circles indicate sulfate transporter and PAPS transporter, respectively. Cytosolic ATP sulfurylase (*cyt-ATPS2*) forms by alternative translational initiation (Bohrer et al., 2014). Abbreviations of metabolites: APS, adenosine-5′-phosphosulfate; Cys, cysteine; GLS, glucosinolate; GSH, glutathione; Met, methionine; PAP, 5′-phosphoadenosine 3′-phosphate; PAPS, 3′-phosphoadenosine 5′-phosphosulfate. Abbreviations of enzymes and transporters: *APK*, APS kinase; *APR*, APS reductase; *ATPS*, ATP sulfurylase; *FRY1*, inositol polyphosphate 1-phosphatase (*FIERY1*); *PAPST1*, PAPS transporter; *SiR*, sulfite reductase; *SOT*, sulfotransferase; *SULTR*, sulfate transporter.

Sulfate assimilation occurs in both plastids and cytosol in vascular plants (**Figure 1**; reviewed in Takahashi et al., 2011; Gigolashvili and Kopriva, 2014; Koprivova and Kopriva, 2014). Sulfate imported across the plasma membrane is the primary substrate provided to the sulfate assimilation pathways, where the ATP sulfurylase (ATPS) serves as an enzyme to catalyze the initial metabolic reaction generating adenosine 5′-phosphosulfate (APS) from ATP and sulfate in both plastids and cytosol. APS is subsequently phosphorylated to 3′-phosphoadenosine 5′-phosphosulfate (PAPS) by the APS kinase (APK), or reduced to sulfite through the function of the APS reductase (APR). APK is present in both plastids and cytosol for phosphorylation, while APR and the subsequent pathway enzyme, sulfite reductase (SiR), are localized only in plastids for catalyzing the reduction steps. The sulfate assimilation pathway thus bifurcates into two directions to phosphorylate or reduce APS in plastids, whereas only the APS phosphorylation pathway is present in cytosol (**Figure 1**).

Plants switch-control the APS reduction and phosphorylation pathways to change the partitioning of sulfur into the primary and secondary metabolisms (Kopriva et al., 2012). APR plays a key role in channeling APS into the sulfate reduction pathway, responding to the demands for Cys and GSH. The demand-driven flux control mechanism has been suggested based on observations of APR transcripts and proteins over-accumulating following sulfate deprivation and being repressed in the presence of reduced S sources (Takahashi et al., 1997; Vauclare et al., 2002). The significance of APR in the primary sulfur metabolism is evidenced by the accumulation of sulfate and total S in *Arabidopsis* accessions with less active variants of APR2 enzyme (Loudet et al., 2007; Chao et al., 2014). In contrast, the expression of plastidic APK, which is required for PAPS biosynthesis providing sulfate donors used in secondary sulfur metabolism, is repressed under sulfur-deficient conditions (Maruyama-Nakashita et al., 2006; Mugford et al., 2009). In addition to the transcriptional mechanisms, APR and APK enzyme activities are regulated by the redox status as demonstrated by *Arabidopsis* APR1 and APK1 gaining maximum catalytic efficiency in their oxidized and reduced forms, respectively (Bick et al., 2001; Ravilious and Jez, 2012; Ravilious et al., 2012, 2013). Thus, the metabolic flux through the bifurcate pathway for APS utilization is affected by both transcriptional and post-transcriptional mechanisms controlling enzyme activity.

## INTEGRATION OF PAPS METABOLISM THROUGH PAPS TRANSPORTER

The plastidic and cytosolic pathways merge following PAPS biosynthesis as PAPS is mainly utilized in the cytosol (**Figure 1**) where it serves as sulfate donor for synthesizing sulfated metabolites including glucosinolates (Klein and Papenbrock, 2004; Piotrowski et al., 2004; Hirai et al., 2005). Therefore, PAPS metabolism has to include a PAPS transporter in the plastid envelopes. Indeed, a PAPS transporter (PAPST1) has been found to export PAPS from plastids to cytosol (Gigolashvili et al., 2012). When sulfotransferases (SOT) synthesize sulfated metabolites, the sulfate moieties of PAPS are transferred to the hydroxyl groups of suitable acceptors, and 3′-phosphoadenosine 5′-phosphate (PAP) is generated as a byproduct. PAP is, however, a cytotoxic compound, as it inhibits RNA metabolizing enzymes responsible for decomposing aberrant RNA (Gy et al., 2007). Since PAPST1 is capable of facilitating the plastid import of PAP to be coupled with the export of PAPS, PAP can be degraded to AMP by PAP phosphatase, FIERY1 (FRY1), in plastids (Rodríguez et al., 2010; Estavillo et al., 2011). PAPS biosynthesis, PAPS utilization, and PAP degradation are therefore connected as a sequential network of metabolic steps in both plastids and cytosol. Such functional interplays can be achieved only with the presence of a suitable transporter, such as PAPST1, that enables the PAPS/PAP exchange following the concentration gradients of substrates necessarily formed across the plastid envelope (**Figure 1**).

The absence of FRY1 induces plant responses to drought, salinity, cold, and excess light stresses, where ABA and jasmonate are involved in signaling (Xiong et al., 2001; Wilson et al., 2009; Rodríguez et al., 2010; Chen et al., 2011; Estavillo et al., 2011; Chan et al., 2013). The significance of the FRY1-mediated pathway lies in the fact that PAP can be a retrograde signal for inducing molecular mechanisms protecting viable cells from stresses in adverse environments. Given the pathway connections with the PAPS/PAP exchange across the plastid envelope (Gigolashvili et al., 2012), cellular PAP concentrations are likely modulated by FRY1 in plastids (Rodríguez et al., 2010; Estavillo et al., 2011). Chloroplast-mitochondrion dual localizations of FRY1 from *Arabidopsis* and PAPST from rice further suggest that mitochondria also serve for detoxifying PAP (Estavillo et al., 2011; Xu et al., 2013). Cross-species conservation of PAP metabolism requires further investigation.

## FLUX CONTROL

In this framework of metabolic pathway connections, abundance and functions of ATPS and APK in plastids and cytosol, together with efficiency of PAPS transport, appear pivotal for controlling subcellular distributions of PAPS and PAP (**Figure 1**). ATPS and APK are predominant in plastids, as evident from the localization of individual isoforms, distribution of enzyme activities, and phenotypes of corresponding mutants (Rotte and Leustek, 2000; Mugford et al., 2009). Such subcellular distributions of ATPS and APK may be important to increase the concentration gradient of PAPS across the plastid envelope, leading to an increased export of PAPS from the plastids to the cytosol as well as a more efficient transport of PAP into the plastids for its detoxification to be accomplished. Since SOTs and consequently PAP production are localized in the cytosol (**Figure 1**), increase in PAPS synthesis in the cytosol would thus prevent PAP/PAPS shuttling and may lead to accumulation of PAP in the cytosol.

These flux control models are supported in part by evidence showing a strong requirement of plastidic APK for synthesis of sulfated metabolites including glucosinolates (Mugford et al., 2009). The phenotypes of the *Arabidopsis apk1 apk2* mutant also suggest that cytosolic APK3 is not compensating for the loss of plastidic APK activity to provide sulfate donors to SOT (Mugford et al., 2009). Mutants in PAPST1 present similar phenotypes with accumulation of desulfo-precursors of sulfated compounds, pointing to the importance of the transporter, however, as these phenotypes are milder than those of *apk1 apk2* plants, another PAPST1 has to be postulated (Gigolashvili et al., 2012). It is therefore conceivable that APK, PAPST1, and SOT are functionally coupled to utilize PAPS and sequester PAP to the plastids. In this metabolic cycle, the PAPS/PAP shuttling mechanism may not properly operate when the plastids are deficient in PAPS. To avoid elevation of PAP concentration in the cytosol, the sulfation reactions catalyzed by SOTs may be not only limited by low PAPS supply, but also actively inhibited when the PAPS/PAP shuttling mechanism is disabled. The coordinate induction of PAPST1, SOT, and FRY1 gene expression in the *apk1 apk2* mutant suggests that a transcriptional coexpression mechanism is activated in an attempt to overcome the defect in plastidic PAPS production (Mugford et al., 2009). In contrast, PAP accumulates disproportionately in the plastids of *fry1/fou8* mutants (Estavillo et al., 2011; Lee et al., 2012), leading to disturbance of the PAP gradient across plastid envelopes. Such circumstances also appear unfavorable for PAPST1 to shuttle PAPS/PAP and to cooperate with APK and SOT to produce sulfated metabolites (Lee et al., 2012).

The overexpression of a bacterial APK in *Arabidopsis* in either plastids or cytosol demonstrates, however, rather complex metabolic interconnections (Mugford et al., 2011). In the APK-overexpressing lines, APS is more likely used for synthesizing PAPS than producing sulfite. Limitation of PAPS availability can induce the expression of glucosinolate biosynthetic genes (Mugford et al., 2009). In contrast, an increased supply of PAPS does not seem to have an opposing effect on gene expression in glucosinolate biosynthesis but rather induces accumulation of *MAM3* and *SOT17* transcripts in the APK-overexpressing lines (Mugford et al., 2011). Furthermore, overexpression of APK causes no significant effect on increasing the flux of glucosinolate production, suggesting that pathways are under control of multifaceted mechanisms. In contrast, the metabolic flux of reductive sulfate assimilation appears to increase for adjustment of Cys and GSH biosynthesis in the APK-overexpressing lines (Mugford et al., 2011). It is possible to hypothesize that APS can be limiting in APK overexpressors, and its shortage may trigger an increase in metabolic flux of reductive sulfate assimilation. Metabolic regulation by APS has been described in bacteria (Bykowski et al., 2002).

APS biosynthesis is a thermodynamically unfavorable reaction and can be a bottleneck of the sulfate assimilation pathway. With regard to the metabolic flux control through the function of ATPS, plastidic ATPS1 makes substantial contribution to the reductive sulfate assimilation pathway (i.e., Cys and GSH biosynthesis) in *Arabidopsis* (Kawashima et al., 2011). In support of this evidence, *ATPS1* is found as a genetic locus that significantly affects sulfate accumulation among the *Arabidopsis* natural variations (Koprivova et al., 2013). Furthermore, a reaction mechanism of substrate-enzyme interaction is proposed based on structural and kinetic analyzes of ATPS1 (Herrmann et al., 2014). In contrast to a wealth of information documenting the function and regulation of plastidic ATPS1, the genetic identity of a cytosolic isoform has remained elusive until recently, when alternative translational initiation of ATPS2 has been identified as a potential mechanism underlying the cytosolic ATPS activity in *Arabidopsis* (Bohrer et al., 2014). It has been reported that cytosolic ATPS activity becomes relatively abundant in matured *Arabidopsis* leaves (Rotte and Leustek, 2000), suggesting that the presence of cytosolic isoform may be conditional. Identification of mechanisms involved in regulation of cytosolic ATPS isoform will open a way to altering the function of this key enzyme and engineering metabolic flux partitioning of sulfate assimilation.

## CONCLUSION

Sulfur metabolic enzymes are not equally expressed in all plant cell types and organelles. ATPS, APK, APR, and PAPST focused on in this article, represent a sub-network of sulfur assimilation pathway, in which partitioning between cytosol and plastids is particularly important. Details of the subcellular localizations of individual isoforms have only recently been acquired and although the identity of at least one additional PAPST1 is still not known, this knowledge will facilitate the dissection of isoform-dependent and compartment-specific functions of these enzymes and transporters and provide new insights into their contributions to control of flux through sulfate assimilation. Given the biological significance of metabolites synthesized in this pathway, the flux regulation of APS/PAPS biosynthesis in specific cell-types and compartments may be associated with physiological adaptations.

## Conflict of Interest Statement

The Reviewer Stephan Krueger declares that, despite having collaborated in the past with the author Stanislav Kopriva, the review process was handled objectively and no conflict of interest exists. The authors declare that the research was conducted in the absence of any commercial or financial relationships that could be construed as a potential conflict of interest.
